# Gas Atmosphere Innovation Applied to Prolong the Shelf Life of ‘Regina’ Sweet Cherries

**DOI:** 10.3390/plants14152440

**Published:** 2025-08-06

**Authors:** Rodrigo Neira-Ojeda, Sebastián Rodriguez, Cristian Hernández-Adasme, Violeta Muñoz, Dakary Delgadillo, Bo Sun, Xiao Yang, Victor Hugo Escalona

**Affiliations:** 1 Centro de Estudios de Postcosecha, Facultad de Ciencias Agronómicas, Universidad de Chile, La Pintana, Santiago 8820808, Chile; rodrigo.neira@uchile.cl (R.N.-O.); s.rodriguez.4@ug.uchile.cl (S.R.); criherna@ug.uchile.cl (C.H.-A.); violeta.munoz@uchile.cl (V.M.); dakary.delgadillo@uchile.cl (D.D.); 2 College of Horticulture, Sichuan Agricultural University, Chengdu 611130, China; bsun@sicau.edu.cn; 3 Institute of Urban Agriculture, Chinese Academy of Agricultural Sciences, Chengdu National Agricultural Science and Technology Center, Chengdu 610000, China; yangxiao@caas.cn

**Keywords:** controlled atmosphere, modified atmosphere, postharvest, browning, fruit quality, prolonged storage, maturity stages

## Abstract

In this study, the impact of moderate and high CO_2_ and O_2_ levels was compared to low and moderate gas combinations during prolonged storage on the quality of Regina sweet cherries harvested in different maturity stages, particularly in terms of decreasing internal browning. Fruits were harvested in two different maturity stages (Light and Dark Mahogany skin color) and stored in CA of 15% CO_2_ + 10% O_2_; 10% CO_2_ + 10% O_2_; 10% CO_2_ + 5% O_2_; 5% CO_2_ + 5% O_2_ and MA of 4 to 5% CO_2_ + 16 to 17% O_2_ for 30 and 40 days at 0 °C and 90% RH, followed by a marketing period. After the storage, both maturity stages significantly reduced internal browning, decay, and visual quality losses in CA with 10–15% CO_2_ and 10% O_2_. In addition, it preserved luminosity, total soluble solids (TSSs), titratable acidity (TA), and bioactive compounds such as anthocyanins and phenols. This treatment also maintained the visual appearance of the sweet cherries, favoring their market acceptance. At the same time, the light red fruits showed a better general quality compared to darker color after the storage. In conclusion, a controlled atmosphere with optimized CO_2_ and O_2_ concentrations, together with harvesting with a Light Mahogany external color, represents an effective strategy to extend the shelf life of Regina sweet cherries up to 40 days plus the marketing period, maintaining their physical and sensory quality for export markets.

## 1. Introduction

Sweet cherries (*Prunus avium* L.) are non-climacteric fruits with a moderate respiration rate (10 to 20 mg CO_2_ kg^−1^ h^−1^ at 0 °C) and very low ethylene production [1]. Despite this, they are highly perishable due to rapid dehydration of the pedicel and fruit, followed by loss of firmness, acidity and development of decay [2]. To delay deterioration, immediate chilling to temperatures of −1 to 0 °C and relative humidity above 95% is recommended, allowing optimal quality for 2 to 4 weeks [1].

In recent years, Chile has established itself as the most important cherry exporter in the southern hemisphere, reaching a shipment volume of 529 thousand tons, which represents 59% of the world market in 2024/25. The main Chilean exported cultivars were Lapins (37%), Santina (31%), and Regina (14%). China stood out as the main Chilean cherry destination market, receiving 91% of Chilean exports [3].

The Regina cultivar is characterized by its sweet flavor, resistance to mechanical damage, and ease of handling due to its long pedicel [4]. However, this cultivar faces important postharvest limitations in periods longer than 30 days of storage, showing loss of firmness, internal browning, and flavor reduction. These limitations are especially critical in Chilean exports to far markets such as Asia, where sea transport can take 25 to 40 days. Even this time may increase due to eventualities during transport or in case of health emergencies.

Internal browning is responsible for about 50% of losses in pre- and post-harvest handling of fresh produce. This disorder is caused by the activity of oxidative enzymes such as polyphenoloxidase (PPO), which produce an unattractive brown color when cell membrane integrity is disturbed [5]. In the Regina cultivar, the incidence of internal browning can reach levels ranging from 13% to 42% after 45 days of storage [6].

Changing the gas composition by using controlled or modified atmosphere (CA and MA) techniques can be useful to reduce metabolic activity and maintain quality in sweet cherries, reducing dehydration and the development of decay under specific oxygen and carbon dioxide concentrations as long as temperature conditions remain optimal [2,7]. In sweet cherries, high concentrations of CO_2_ (10 or 20%) help reduce rot and maintain fruit firmness, acidity, and color. Recommended concentrations in this species are usually between 10–15% CO_2_ and 3–10% O_2_ [1]. Previous studies have shown that a combination of 10% CO_2_ + 5% O_2_ + 85% argon in varieties such as Lapins allowed for preserving quality for 63 days at 0 °C and high relative humidity, reducing softening, internal browning, and lipid peroxidation [8]. Similarly, Remón et al. [9] showed that in the Sweetheart cultivar stored in CA of 2% CO_2_ and 5% O_2_, there was reduced PPO activity, stabilizing anthocyanin content compared to air-conditioned storage at 1 °C. However, O_2_ levels below 1% can cause fermentation, and CO_2_ levels above 30% cause external browning in the Bing cultivar [2].

It is suggested that the application of high concentrations of CO_2_ combined with moderate levels of O_2_ represents an effective strategy for maximizing the beneficial effects of CO_2_ during the storage of sweet cherries, while preventing the onset of unwanted fermentation that compromises fruit quality. This gas combination not only optimizes the performance of refrigerated containers but also improves internal storage conditions, significantly extending the product’s shelf life. This strategy is particularly relevant in the fruit sea transport industry, where sweet cherries are often exported in refrigerated shipping containers adapted to use CA. Therefore, containers can get an airtight enough seal to reach moderate and high CO_2_ levels, but not very low O_2_ levels. Considering that and references mentioned that low and moderate CO_2_ or O_2_ levels (3 to 10%) are the most recommended for other sweet cherries cultivars such as Lapins, Bing, or Sweetheart [1,9,10], this new study evaluated the use of moderate to high CO_2_ plus low and moderate O_2_ compared to the normal gas combinations recorded in MA of 5 to 6% CO_2_ plus 14 to 15% O_2_, which suggests its potential effectiveness for the Regina cultivar.

It was also considered essential to include the factor of maturity stage at harvest, given that this variable directly influences the physiological response of the fruit and the evolution of its quality during storage in a controlled atmosphere [1,11,12]. Although it is recognized that maturity affects postharvest behavior, there are only a few studies that analyze how different maturity stages interact with the effectiveness of controlled atmospheres to preserve the overall quality of Regina. The evaluation of different commercial maturity stages must consider the consumer’s perspective because darker color is a valuable visual attribute that directly influences the purchase decision [13], but this color can also be related to senescent fruit with shorter postharvest storage potential. In this context, the objective of this study is to evaluate the effect of different gas compositions on the quality of Regina sweet cherries harvested at two maturity stages and stored under refrigeration for up to 40 days, contributing to the development of strategies that optimize their preservation for export markets.

## 2. Results

### 2.1. Pedicel Dehydration and Decay

No interactions between the factors or significant differences in pedicel dehydration were observed in any of the measurements. The average percentage of fruit without dehydration (W/PD) was 95.6% at the beginning of storage and 92.5% and 87.3% after 30 and 40 days of storage, plus the marketing period.

Regarding the incidence of decay, no significant differences were found between maturity stages at the beginning of storage, with an average of 0.2%. However, in both storage times, significant differences were observed depending on the atmosphere used. Treatments with more than 10% CO_2_ showed a significant effect in reducing the incidence of decay, achieving reductions of 91% compared to the MA-4 + 16 after both storage periods (Table 1).

### 2.2. Browning Index (BI)

No internal browning was observed in the sweet cherries at the beginning of storage. However, after 30 days of storage, plus the marketing period, differences were identified in the gas atmosphere factor, with a positive effect under 10 + 10 with 29% less IB compared to the MA (Table 2). After 40 + 4 days, significant differences were found between the factors independently. In the maturity stage factor, the highest value was recorded in DM, with 19% more BI compared to LM. For gas atmosphere factor, treatments with moderate to high CO_2_ (10 and 15%) showed 36% less IB compared to MA of 4+16 (Table 2).

### 2.3. Firmness

#### 2.3.1. Texture Analyzer Method

At the beginning of storage, no significant differences were observed in the firmness of sweet cherries according to maturity stage. After 30 days of storage plus the marketing period, significant differences were observed between the maturity stages, with LM showing 10% more firmness than DM. Similarly, after 40 + 4 days, significant differences were found in both factors independently. In this regard, LM retained 15% more firmness than DM, while the CA 5 + 5 and CA 15 + 10 treatments showed an increase in firmness of 9 and 12%, respectively, compared to the MA treatment (Table 3).

#### 2.3.2. Durometer Method

Initially, no significant differences were observed between fruits harvested at different stages of maturity. After 30 + 4 days, there were significant differences in both factors independently. In terms of maturity stage, LM showed 9% more firmness compared to DM. In the atmospheres, CA treatments showed the highest values, with up to 4% more firmness compared to the MA treatment. After 40 + 4 days, 10 + 5 and 10 + 10 reached similar firmness to MA, and the lowest were found in 10 + 5 and 15 + 10 (Table 3).

### 2.4. Internal Flesh Color

**Figure 1:**
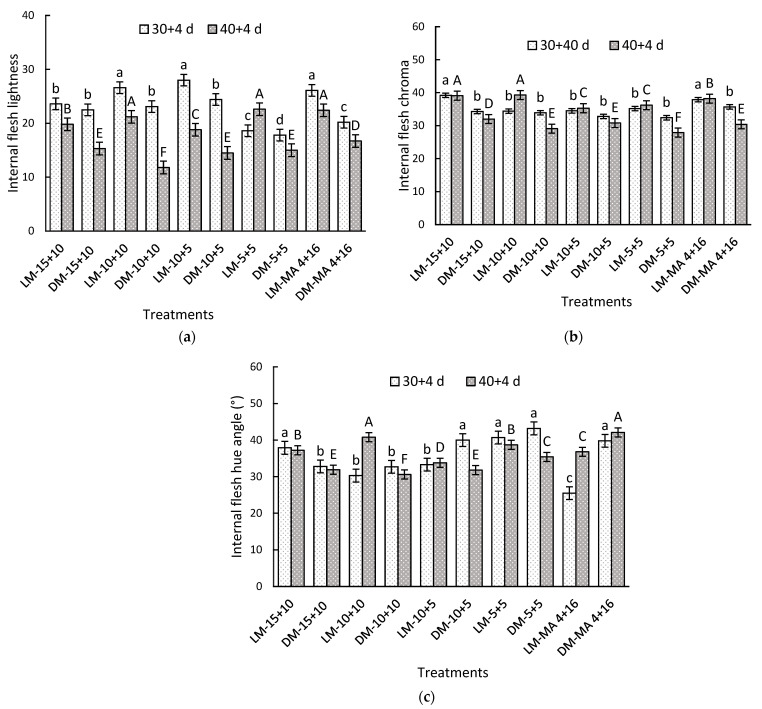
Internal flesh color parameters of Regina sweet cherries (**a**) lightness, (**b**) chroma, and (**c**) hue angle, between the interaction of maturity stage and gas atmosphere factors. LM: Light Mahogany, DM: Dark Mahogany; CO_2_ (%) + O_2_ (%): 15 + 10, 10 + 10, 10 + 5, 5 + 5, 4 + 16. Different lowercase letters indicate statistically significant differences at 30 days at 0 °C + 2 days at 5 °C + 2 days at 10 °C (30 + 4), and different capital letters indicate statistically significant differences at 40 days at 0°C + 2 days at 5 °C + 2 days at 10 °C (40 + 4) respectively, according to Fisher’s multiple comparison test (*p*-value < 0.05). Values present mean ± standard error.

At the beginning of storage, LM fruits exhibited significantly higher values of lightness, chroma, and hue angle compared to DM fruits (Table 4). After 30 + 4 days, significant interaction between factors was observed in all parameters evaluated. In terms of lightness, LM fruits under 10 + 10 (26.6), 10 + 5 (28.0), and MA 4 + 16 (26.1) showed the highest values compared to DM-5 + 5, which recorded a value of 17.8 (Figure 1a). LM fruits treated with CA maintained up to 60% higher hue angle values compared to the control (MA) treatment (Figure 1c). After 40 + 4 days, the LM-15 + 10 and LM-10 + 10 treatments exhibited the highest chroma values, with 39.11 and 39.26, respectively, while the lowest values were observed in DM-5 + 5, which recorded 27.9 (29% lower). During this period, the highest lightness and chroma values were observed in the LM maturity stage treatments compared to DM fruit. However, in most treatments, internal lightness decreased as storage time increased (Figure 1b).

### 2.5. External Skin Color

**Figure 2:**
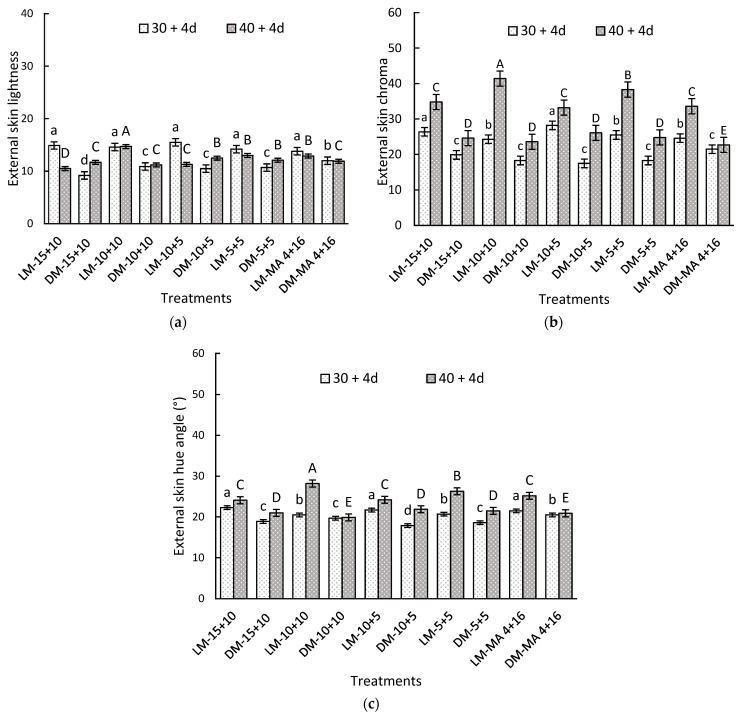
External skin color parameters of Regina sweet cherries (**a**) lightness, (**b**) chroma, and (**c**) hue angle, between the interaction of maturity stage and gas atmosphere factors. LM: Light Mahogany, DM: Dark Mahogany; CO_2_ (%) + O_2_ (%): 15 + 10, 10 + 10, 10 + 5, 5 + 5, 4 + 16. Different lowercase letters indicate statistically significant differences at 30 days at 0 °C + 2 days at 5 °C + 2 days at 10 °C (30 + 4), and different capital letters indicate statistically significant differences at 40 days at 0 °C + 2 days at 5 °C + 2 days at 10 °C (40 + 4) respectively, according to Fisher’s multiple comparison test (*p*-value < 0.05). Values present mean ± standard error.

In the initial evaluation, the skin color of LM maturity exhibited significantly higher values for lightness, chroma, and hue angle compared to DM fruits (Table 4). The differences observed between maturity stages were maintained throughout the storage period. The initial lightness values were 32.04 for LM and 26.98 for DM. This parameter showed a marked decrease after 30 + 4 days, reaching values below 14.9 in LM and 12.0 in DM. After 30 and 40 days of storage, followed by the marketing period, a significant interaction between the evaluated factors was observed. The LM treatments showed the highest values for L*, C*, and H* compared to DM (Figure 2) in both storage evaluation periods.

On day 30 + 4, DM fruits stored in MA showed 30 and 15% more lightness and hue angle compared to the DM in CAs (Figure 2a,b). A significant decrease in lightness (57 to 66%) in LM and DM indicated an evolution of the external color despite the gas atmosphere used (Figure 2a).

After 40 + 4 days, the LM-10 + 10 treatment recorded the highest values for L* (14.7), C* (41.4), and H* (28.2°). Conversely, the lowest values were found in the LM-15+10 treatment for lightness (10.5), DM-MA 4 + 16 for chroma (22.7), DM-10 + 10 (19.9°), and DM-MA 4 + 16 (20.9°) for hue angle (Figure 2). Overall, it was observed that chroma and hue angle increased with storage time.

### 2.6. Total Soluble Solids (TSSs), Titratable Acidity (TA), and TSS/TA Ratio

#### 2.6.1. Total Soluble Solids (TSSs)

At the beginning of storage, significant differences were observed between maturity stages, where LM and DM recorded 19.70 and 21.33%, respectively (Table 5).

After 30+4 days, a significant interaction was observed between the evaluated factors. LM showed about 15% more TSSs in CA than MA (Table 6).

On day 40 + 4, significant differences were observed for the gas atmosphere factor independently. The lowest value was reached in CA of 10 + 5, 9% lower than MA with 4 + 16 (Table 7). However, in both storage periods, all treatments decreased TSSs compared to the initial values.

#### 2.6.2. Titratable Acidity (TA)

At the beginning of storage, LM registered 22% higher TA than DM; however, this difference was not maintained during storage (Table 5, Table 6 and Table 7). After the storage, CA was more effective in preserving acidity than MA.

After 30 + 4, a significant interaction was observed between both factors. For LM fruits, CA of 10 to 15% CO_2_ plus 10% O_2_ registered 7% more acidity than MA with 4 + 16. DM fruits stored in CA-15 + 10 maintained higher acidity levels (about 35%) than MA-4 + 16 (Table 6).

After 40 + 4 days, significant differences were found for the gas atmosphere factor independently, with no differences between maturity stages. The highest values (over 23%) were recorded in CA compared to MA (Table 7).

#### 2.6.3. TSSs/TA Ratio

Initially, TSSs/TA showed a significant difference between both maturity stages, being 33% lower LM than DM (Table 5). Throughout the storage, a significant increase was observed on day 30 + 4 for all treatments, and an interaction between the factors kept a lower ratio for LM treatments. In fact, LM-15 + 10 reached 28.8, 62% lower value compared to DM-MA 4 + 16 (46.7) (Table 6). After 40 + 4 days, significant differences were observed in both factors independently. LM registered values 11% lower than DM, while CAs of 10 + 10 and 10 + 5 achieved values up to 30% lower than the MA of 4 + 16 (Table 7). In general, CA with high CO_2_ levels was more effective in conserving TSSs and TA values.

### 2.7. Biochemical Parameters

#### 2.7.1. Total Anthocyanin Concentration (TAC)

**Figure 3:**
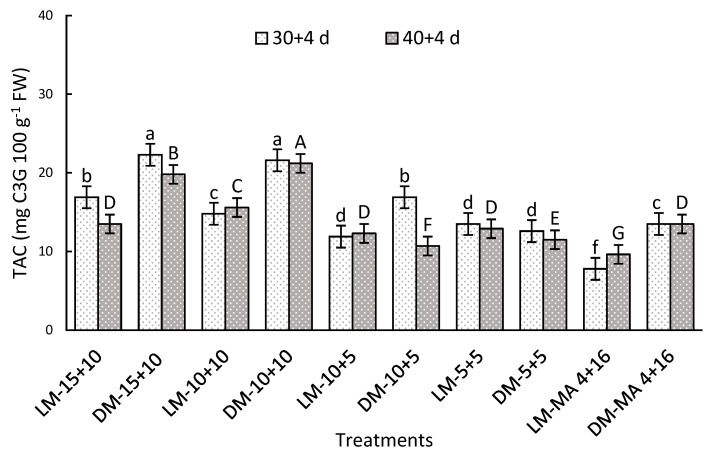
Total anthocyanin concentration (TAC) (mg C3G 100 g^−1^ FW) in Regina sweet cherries after storage, analyzed based on the interaction between factors. LM: Light Mahogany, DM: Dark Mahogany; CO_2_ (%)+O_2_ (%): 15 + 10, 10 + 10, 10 + 5, 5 + 5, 4 + 16. Different lowercase letters indicate statistically significant differences at 30 days at 0 °C + 2 days at 5 °C + 2 days at 10 °C (30 + 4), and different capital letters indicate statistically significant differences at 40 days at 0 °C + 2 days at 5 °C + 2 days at 10 °C (40 + 4) respectively, according to Fisher’s multiple comparison test (*p*-value < 0.05). Values present mean ± standard error.

A significant interaction was observed between the factors evaluated during both storage periods. After 30 + 4 days, the highest TAC values were recorded in DM in 15 + 10 and 10 + 10, being 65 and 185% higher than DM or LM in MA 4 + 16, respectively (Figure 3). On day 40 + 4, DM stored in 15 + 10 or 10 + 10 showed 57 and 120% higher values than DM or LM in MA 4 + 16, respectively (Figure 3). In general, TAC was higher in DM than LM particularly in atmospheres with moderate and high CO_2_ levels (10 or 15%).

#### 2.7.2. Total Polyphenol Concentration (TPC)

**Figure 4:**
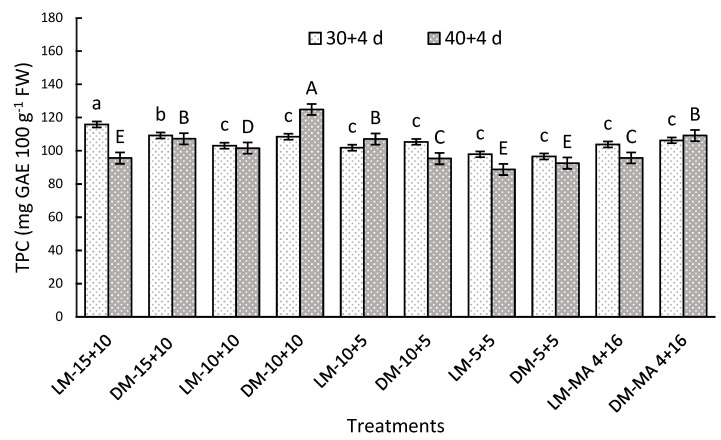
Total phenolic concentration (TPC) (mg GAE 100 g^−1^ FW) in Regina sweet cherries after storage, analyzed based on the interaction between factors. LM: Light Mahogany, DM: Dark Mahogany; CO_2_ (%) + O_2_ (%): 15 + 10, 10 + 10, 10 + 5, 5 + 5, 4 + 16. Different lowercase letters indicate statistically significant differences at 30 days at 0 °C + 2 days at 5 °C + 2 days at 10 °C (30 + 4), and different capital letters indicate statistically significant differences at 40 days at 0 °C + 2 days at 5 °C + 2 days at 10 °C (40 + 4) respectively, according to Fisher’s multiple comparison test (*p*-value < 0.05). Values present mean ± standard error.

Maturity stages and gas atmosphere had significant interaction for both storage periods. After 30 + 4 days, CA of 15 + 10 showed 12% higher TPC in both maturity stages, compared to MA-4 + 16 (Figure 4). After 40 + 4 days, the DM-10 + 10 reached high TPC of 124.95 mg GAE 100 g^−1^ FW in relation to DM-MA 4 + 16 (109.2 mg GAE 100 g^−1^ FW) (Figure 4).

#### 2.7.3. Antioxidant Capacity by DPPH Method

**Figure 5:**
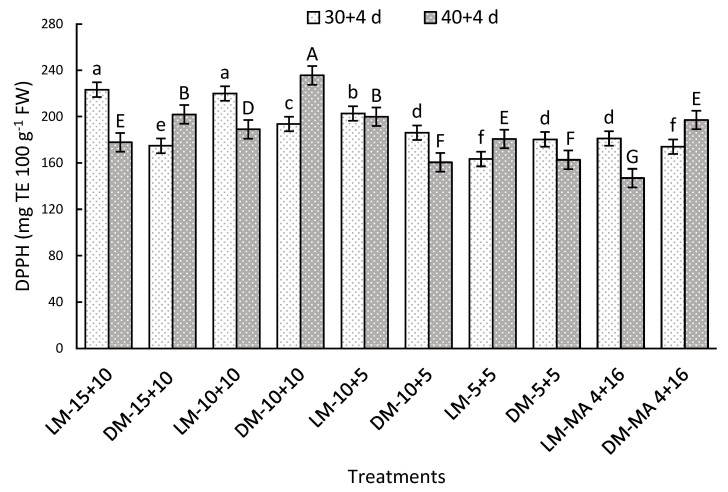
Antioxidant capacity by DPPH method (mg TE 100 g^−1^ FW) in Regina sweet cherries after storage, analyzed based on the interaction between factors. LM: Light Mahogany, DM: Dark Mahogany; CO_2_ (%) + O_2_ (%): 15 + 10, 10 + 10, 10 + 5, 5 + 5, 4 + 16. Different lowercase letters indicate statistically significant differences at 30 days at 0 °C + 2 days at 5 °C + 2 days at 10 °C (30 + 4), and different capital letters indicate statistically significant differences at 40 days at 0 °C + 2 days at 5 °C + 2 days at 10 °C (40 + 4) respectively, according to Fisher’s multiple comparison test (*p*-value < 0.05). Values present mean ± standard error.

A significant interaction was observed between the factors after both storage periods. On day 30 + 4, LM stored in 15 + 10 or 10 + 10 reached 223.32 or 219.90 mg TE 100 g^−1^ FW, respectively being 23% higher than LM-MA 4 + 16 (Figure 5). After 40 + 4 days, the most notable treatments were DM-10 + 10 and DM-15 + 10 with 235.6 and 201.9 mg TE 100 g^−1^ FW, while the lowest value was recorded in LM-MA 4 + 16 (147.0 mg TE 100 g^−1^ FW) (Figure 5). These results show a positive effect of CO_2_ concentrations above 10% on the preservation of the antioxidant capacity of Regina cv.

#### 2.7.4. Antioxidant Capacity by FRAP Method

**Figure 6:**
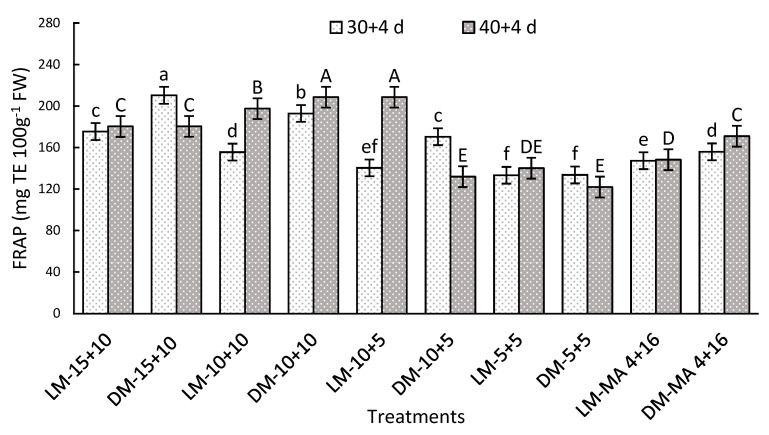
Antioxidant capacity by FRAP method (mg TE 100 g^−1^ FW) in Regina sweet cherries after storage, analyzed based on the interaction between factors. LM: Light Mahogany, DM: Dark Mahogany; CO_2_ (%)+O_2_ (%): 15 + 10, 10 + 10, 10 + 5, 5 + 5, 4 + 16. Different lowercase letters indicate statistically significant differences at 30 days at 0 °C + 2 days at 5 °C + 2 days at 10 °C (30 + 4), and different capital letters indicate statistically significant differences at 40 days at 0 °C + 2 days at 5 °C + 2 days at 10 °C (40 + 4) respectively, according to Fisher’s multiple comparison test (*p*-value < 0.05). Values present mean ± standard error.

After storage, a significant interaction between the factors was observed. On day 30 + 4, DM stored in moderate to high CO_2_ levels of 10–15% improved by 35% the values compared to MA-4 + 16, while darker fruits showed the highest antioxidant capacity (Figure 6). After 40 + 4 days, LM-10 + 5 and DM-10 + 10 recorded the highest values of 208.51 and 208.45 mg TE 100 g^−1^ FW), exceeding LM-MA 4 + 16 by 41% (Figure 6).

## 3. Discussion

### 3.1. Pedicel Dehydration and Decay

Consumer acceptability of sweet cherries depends on several visual characteristics, particularly a green pedicel and the absence of internal rot and browning [8,14]. The results showed that storage of Regina sweet cherries at 0 °C for 30 and 40 days, followed by a marketing period, and the use of controlled atmosphere (CA) with high gas concentrations (more than 5% CO_2_ + 10% O_2_) significantly reduced the incidence of decay. Specifically, the use of CA with 10% or 15% CO_2_ for 30 days reduced the decay rate to almost 0%. Similar results have been reported for rot reduction in Lapins sweet cherries stored at 0 °C and 95% relative humidity under CA 10 + 5 [8]. This antifungal effect of CO_2_ concentrations higher than 10% and/or low O_2_ concentrations is supported in the literature, since phytopathogenic fungi require O_2_ for their survival and development [2,15]. The mechanism of action of high levels of CO_2_ on aerobic fungi is not yet fully understood, although it is believed to have an effect on cell membrane phospholipids and a decrease in extracellular and intracellular pH, which would inhibit their growth [16].

### 3.2. Browning Index (BI)

Internal browning is an enzymatic process mediated by polyphenol oxidases (PPOs), which are activated by stress or senescence. These enzymes oxidize phenolic compounds in the presence of oxygen, generating brown pigments that negatively affect fruit quality [17,18]. This phenomenon is closely related to respiration and the production of reactive oxygen species (ROSs), which damage cell structures and accelerate fruit senescence. Several studies have shown that oxygen reduction in the storage environment decreases both oxidation and ROS synthesis, thus extending fruit shelf life [5,19,20]. Reducing oxygen from 21% (atmospheric level) to 10% slows respiration rates and prolongs fruit shelf life without inducing anaerobic conditions, although oxygen levels as low as 5% increase the risk of fermentation, resulting in the production of compounds such as ethanol and acetaldehyde that negatively affect sensory quality [21]. Storage conditions with 10% CO_2_ and 5% O_2_ have been shown to be optimal for maintaining the quality of sweet cherries by reducing decay, limiting PPO activity, and mitigating oxidative stress [7,22,23]. Our study confirms that sweet cherries stored under controlled atmosphere (CA) with combinations of 10 or 15% CO_2_ + 10% O_2_ exhibited a significant reduction in internal browning after 30 and 40 days of storage, plus the marketing period. These results are in agreement with previous research highlighting how increasing CO_2_ from atmospheric levels (~0.04%) to moderate levels (10–15%) slows respiration, prolongs shelf life, and delays senescence. However, excessive CO_2_ levels (>20%) are associated with severe damage, such as discoloration and sensory deterioration [21]. Our study identified significant differences in susceptibility to internal browning as a function of maturity stage at harvest. After 40 days of storage, sweet cherries at the Dark Mahogany stage showed significantly greater internal browning compared to those at the Light Mahogany stage. This behavior is related to a higher initial metabolic activity in the more mature fruits, which makes them more prone to oxidative stress and accelerates their senescence. These conditions increase the activity of enzymes such as PPOs, intensifying cell deterioration [21,23]. In contrast, fruits harvested at the LM maturity stage showed greater resistance to damage during prolonged storage, which is consistent with previous research linking their lower free phenolic content and lower respiratory rate to greater postharvest stability [24,25]. Therefore, both the maturity stage at harvest and storage conditions are crucial factors in minimizing the incidence of browning and preserving the quality of sweet cherries.

### 3.3. Firmness

Firmness is one of the most critical quality attributes for consumer acceptance when purchasing fruit [12,26,27]. In this study, firmness decreased inversely with storage duration, aligning with previous research on the Lapins cultivar [8]. Fruit harvested at the Light Mahogany stage showed significantly higher compressive strength in all evaluations compared to fruit at the Dark Mahogany stage, consistent with findings reported by Zhang et al. [28] on several varieties. This is attributed to less mature fruits having a more compact cell structure and less degraded cell walls, providing greater rigidity and resistance to stress during storage [29]. In contrast, riper fruit lose firmness more rapidly due to a higher rate of water loss, which accelerates cell wall degradation, reduces cell-cell adhesion, and promotes softening [30]. After 40 days of storage plus the marketing period, treatments MA-4 + 16 and CA 10+10 showed the highest firmness values. MA reduces water vapor loss by maintaining high relative humidity inside the package and decreasing the vapor pressure deficit (VPD) between the environment and the fruit. These conditions slow cellular dehydration, preserve tissue turgor and elasticity, and result in higher firmness [31,32,33].

### 3.4. Color

The characteristic red color of sweet cherries is directly related to the concentration of anthocyanins in the skin, pigments responsible for the red, blue, purple, and black hues in various horticultural products [7]. As occurred in this study, it has been documented that riper fruits have a higher anthocyanin content [34]. In our results, higher lightness values were observed in less mature fruits and with a decrease during storage. Similar results have been reported for Sciazza and Ferrovia cultivars stored for 15 days at 1 °C and 95% relative humidity [35], as well as for Lapins and Skeena cultivars stored under MA at 0 °C [36] and under CA for Lapins [8]. After 30 days of storage and the marketing period, Dark Mahogany maintained higher flesh lightness under CA with concentrations above 10% CO_2_ and 5% O_2_ and external lightness under MA-4 + 16. After 40 days and the marketing period, the LM-10 + 10 treatment maintained high levels of internal and external lightness. The preservation of fruit lightness under CO_2_ concentrations above 10% aligns with previous findings in Bing sweet cherries, where lightness was maintained under CA with high CO_2_ concentrations [37]. High CO_2_ concentrations combined with reduced O_2_ levels have also been reported to delay browning in various cherry varieties during cold storage, contributing to a longer-lasting attractive appearance [7,38].

Chroma of fruit at the LM decreased in all treatments during the first 30 days of storage, consistent with previous studies under similar conditions [8,39]. However, at the DM, an increase in chroma values in cherry skin was observed during this period. After 40 days of storage, chroma values also increased at both maturity stages in all treatments. This behavior has been documented in the Burlat cultivar, where an initial decrease at 5 days of storage was followed by an increase in chroma [8]. According to Markakis [40], the color stability of anthocyanins is influenced by multiple factors, including pH, temperature, presence of enzymes, light, anthocyanin structure and concentration, and interactions with complexing agents such as flavonoids, phenolic acids, and metals. In this study, controlled atmosphere with concentrations between 10 and 15% CO_2_ significantly increased chroma values after 40 days of storage, which agrees with findings in Lapins cherries stored under controlled atmosphere for 63 days at 0 °C and 95% relative humidity [8]. This result suggests that such CO_2_ concentrations in the conservation atmosphere influence anthocyanin stability and synthesis during prolonged storage. It has been reported in several studies that CO_2_-enriched environments reduce the activity of enzymes responsible for anthocyanin degradation [8,41].

During storage, an increase in the hue angle was observed in most treatments, remaining stable or increasing in some cases until 40 days. Previous studies have shown that the hue angle can remain stable or increase in certain preservation treatments, while it can decrease in others, depending on the method used [7,8,39].

### 3.5. Total Soluble Solids (TSSs), Titratable Acidity (TA), and TSSs/TA Ratio

During the final stage of cherry ripening on the tree, there is an increase in TSSs [42], suggesting that red mahogany sweet cherries harvested at this stage accumulate higher TSS levels. In this study, higher values were observed in more mature fruit (DM), a trend that persisted during the first 30 days of storage and the marketing period. During postharvest, fruits continue to respire, which is essential for maintaining metabolic processes such as cellular repair, molecule synthesis, and regulation of water loss. However, this respiration consumes energy reserves, which, over time, deteriorates fruit quality, manifesting as softening, color changes, loss of acidity, and increased TSSs [43]. It has been shown that TSS tends to increase in the first days of storage but begins to decrease after 15 days due to respiratory metabolism [8,35]. In this study, TSS decreased during storage in all treatments, stabilizing after 40 days. After 30 days of storage, controlled atmospheres in LM fruit maintained higher TSS values compared to DM, suggesting that controlled atmospheres may reduce respiration rates, favoring the retention of soluble compounds. However, this advantage was not maintained after 40 days, indicating that other factors influence TSS stability over longer periods.

Titratable acidity (TA) is a key attribute of fruit organoleptic quality, especially in sweet cherries. Titratable acidity reflects effective acidity and, thus, the flavor, with malic acid being the main component in sweet cherry fruits [35,43]. During storage, TA decreases because organic acids are used as primary substrates in respiration and other metabolic processes [43,44,45]. This study also evidenced a decrease in TA during storage, with LM showing higher acidity compared to DM, although these values were equalized at the end of the 40-day storage and marketing period. The decrease was more pronounced in LM cherries and in the control treatment (MA). However, controlled atmospheres with 15% or 10% CO_2_ and 10% O_2_ significantly delayed TA loss, probably due to reduced respiratory rate and other metabolic processes [36,38,46]. Elevated CO_2_ concentrations have been shown to inhibit the decrease in TA during storage [7,8,37]. In this study, this effect was ratified in combination with the less advanced maturity stage.

The balance between TTSs and TA is a key determinant of flavor in sweet cherries, significantly influencing consumer acceptance [12]. In this study, cherries harvested at the LM presented lower values of the TSSs/TA ratio. During storage, there was a progressive decrease in both TSSs and TA, although TSS tended to stabilize toward the end of the 40-day plus marketing period. Flavor loss during storage was initially attributed to the combined reduction in TSSs and TA; however, at later stages, this deterioration was predominantly influenced by the decrease in TA. Dark Mahogany fruits stored in MA showed higher values of the TSSs/TA ratio at 40 days. This could be explained by their higher maturity at harvest, characterized by higher TSS levels and lower TA, making them more susceptible to metabolic changes induced by less stable gas concentrations compared to controlled atmosphere (CA) treatments. On the other hand, CA treatments with CO_2_ concentrations higher than 5% and O_2_ equal to or higher than 5% were more effective in preserving TSSs and TA, maintaining the sensory quality and characteristic flavor of the fruit during prolonged storage. These results are in agreement with previous studies highlighting that atmospheres enriched with high levels of CO_2_ and low levels of O_2_ are efficient in maintaining TSSs and TA in cherries during prolonged storage [37,47].

### 3.6. Total Anthocyanin Concentration (TAC)

Anthocyanins are water-soluble secondary compounds with antioxidant activity, responsible for the red, blue, purple, and black colors of various fruits and vegetables, such as apples, grapes, sweet cherries, strawberries, and blackberries, among others [48,49]. In red fruits, one of the most evident changes during ripening is color transformation, driven by both anthocyanin biosynthesis and chlorophyll degradation, a process that intensifies in the weeks prior to harvest [11,39]. This process was also observed in the present study. Anthocyanins accumulate mainly in the skin and, to a lesser extent, in the pulp, and their concentration may even increase during storage under appropriate conditions [50]. The maturity stage and the time of harvest are critical factors that determine the anthocyanin content in fruits. Previous studies have shown that darker colored fruits tend to have higher anthocyanin concentrations [51,52,53]. This trend was confirmed in the present study, where fruits at the advanced maturity (DM) stage exhibited higher anthocyanin concentrations throughout the storage period. The results indicate that treatments with CO_2_ concentrations of 10% or 15% presented significantly higher anthocyanin levels compared to other treatments in both storage periods. This effect is controversial, since several studies have shown that the application of CO_2_ to various fruits inhibits the increase of anthocyanins, by affecting biosynthesis, degradation, or both [54].

### 3.7. Total Polyphenol Concentration (TPC)

Polyphenols are secondary metabolites present in fruits and vegetables that play a key role in the color, flavor, and antioxidant capacity of cherries [11,26]. Their concentration is influenced by genetic factors, growing conditions, maturity stage, and postharvest management practices [11,55]. Although Serrano et al. [26] reported that phenolic compounds tend to be more abundant in ripe fruit, this pattern was not observed in the present study, as the TPC at the two maturity stages depended on the concentration of gases in the atmosphere. On the other hand, during storage, it is common to observe an initial increase in phenolic compound content, followed by a decrease due to oxidation catalyzed by the enzyme polyphenol oxidase (PPO), a process that contributes to fruit browning [8,17,18]. This dynamic was not clearly manifested in the results of this study. After 30 + 4 days, the CA 15 + 10 retained the highest levels of polyphenols. Extending storage to 40 + 4 days, treatments with 10% CO_2_ stood out for maintaining higher polyphenol concentrations compared to other treatments. Under controlled atmosphere conditions with high levels of CO_2_ and low levels of O_2_, a significant reduction in PPO activity has been documented, which limits the oxidation of phenolic compounds and reduces browning, allowing greater stability of polyphenols during storage [22,56]. Thus, our results suggest that atmospheres with a concentration of 10% CO_2_ or more could preserve polyphenols, reducing browning during prolonged storage.

### 3.8. Antioxidant Capacity

Antioxidant capacity is defined as the potential of certain compounds to neutralize reactive oxygen species (ROS), unstable molecules that contain oxygen, are highly reactive, and are capable of causing cellular damage known as oxidative stress [57]. Among the most prominent antioxidant compounds are anthocyanins and polyphenols, which have attracted considerable attention due to their protective properties [58]. In this study, antioxidant capacity was evaluated by two complementary methods, as it may vary depending on the composition of the sample and the type of oxidants used in the measurement [59]. The results showed that treatments with CO_2_ concentrations of 10 to 15% maintained higher levels of antioxidant capacity in both storage evaluations. This behavior could be attributed to the higher stability of anthocyanins and lower oxidation of polyphenols under these conditions, as discussed in previous analyses. Previous studies have shown a strong correlation between antioxidant capacity and total phenolic compound concentration, supporting the findings of this study [26].

## 4. Materials and Methods

### 4.1. Plant Material and Experimental Design

Sweet cherries (*Prunus avium* L. cv. Regina) were collected on 21 and 26 December 2022 in a commercial field located in Romeral (Region of Maule, Chile). They were selected in two maturity stages by the external color: Light Mahogany (LM, L*: 32.0, C*: 31.5, and H°: 16.1°) and Dark Mahogany (DM; L*: 27.0, C*: 17.0, and H°: 14.9°). Fruits were harvested early in the morning (8 to 9 A.M.) and immediately transported 2 h to the laboratories of the Centre of Postharvest Study (CEPOC). In the laboratory, the fruits were immersed for 15 s in a Fludioxonil solution at a concentration of 230 g L^−1^ (Scholar 230 SC, Syngenta, Chile) at 5 °C. Fruits were sorted according to the maturity stage and placed in 600 g clamshells and packed in perforated bags (0.9 % perforated area). The clamshells were arranged inside plastic cabins (220 L) provided with CO_2_ and O_2_ gas sensors under controlled atmosphere (CA) system (AtmosFix, HappyAgro, Santiago, Chile). Also, temperature and relative humidity sensors (HI148-1, Hanna Instruments, Nusfalău, Romania) were placed inside the bags. As a control treatment, 1 kg of fruits was packed in modified atmosphere (MA) bags (San Jorge Packaging, Chile). The storage periods were carried out for 30 and 40 days at a temperature of 0.0 ± 0.5 °C and a relative humidity of 90–95%. Subsequently, a marketing period was simulated by 2 days at 5 °C, followed by 2 days at 10 °C (+4 d). The experiment followed a completely randomized design with a 2 × 5 factorial structure. The first factor was the maturity stage determined by the two external colors of the skin (light and dark red). For the second factor, five different gas atmospheres were used (CA of: 15% CO_2_ + 10% O_2_; 10% CO_2_ + 10% O_2_; 10% CO_2_ + 5% O_2_; 5% CO_2_ + 5% O_2,_ and MA of 4 to 5% CO_2_ + 16 to 17% O_2_). In total, 10 treatments were evaluated (Table 8) with 3 replications per treatment. The experimental unit was a clamshell with 600 g or 1 kg of fruit.

### 4.2. Physicochemical Evaluations

The evaluations were carried out at the beginning and after each storage period, followed by a simulated marketing time, considering the following parameters.

#### 4.2.1. Pedicel Dehydration and Decay

Pedicel dehydration was evaluated using a 5-point scale based on pedicel weight loss, expressed as a percentage, and its visual correspondence. Twenty fruits were used per repetition, which were weighed individually at the beginning and end of the storage period to calculate relative weight loss. In addition, a visual assessment was performed using a pedicel dehydration scale, which considers the following ranges: 1 = <1%, 2 = 1–2%, 3 = 2–3%, 4 = 3–4%, and 5 = >4%. Categories 1 and 2 were considered acceptable or without pedicle dehydration (W/PD). The incidence of decay was assessed in 50 random fruits by visual inspection and expressed as a percentage of decayed fruits. Decay was defined as the presence of visible fungal decay and/or fruit collapse, juice exudation, or symptoms of fermentation.

#### 4.2.2. Browning Index (BI)

Browning was assessed on a 5-point scale based on the affected pulp area, where 0 = no browning; 1 = <¼ of the pulp area; 2 = ¼ to ½; 3 = ½ to ¾; and 4 = >¾ of pulp area. The browning index (BI) was calculated using the formula:

BI = ∑ (browning score × number of fruits with that score)/(4 × total fruits) × 100

A higher BI value indicates a higher degree of browning in the sample evaluated.

#### 4.2.3. Firmness

Firmness was measured in the equatorial zone of 20 fruits per repetition at room temperature, by means of a non-destructive compression test. A 24 mm diameter tip was used to perform a 1 mm compression on the fruit. The force exerted on the fruit was recorded with a texture analyzer (FirmPro, Happyvolt, Santiago, Chile), and the results were expressed in units of gf mm^−1^. In addition, firmness was measured with a durometer (Baxlo, Barcelona, Spain) on both equatorial sides of 20 other samples and expressed as Shore Units (°Ush).

#### 4.2.4. Color

External and internal color (L*, C*, and H* parameters) were measured in 15 fruits by repetition with a tristimulus colorimeter (Minolta Chromameter, CM2500d, Osaka, Japan). The external and internal colors were evaluated on both equatorial sides of the skin and the flesh after longitudinally cutting them in half and removing the stone. Three color parameters were used to described the color: Lightness (L*, where 0 represents absolute black and 100 corresponds to pure white), chroma (C*, purity or intensity of the color *) and hue angle (H*, where 0° and 360° correspond to red-purple, 90° to yellow, 180° to green, and 270° to blue) [60].

#### 4.2.5. Total Soluble Solids (TSSs), Titratable Acidity (TA), and TSSs/TA Ratio

Juice from 10 fruits per replicate was used for these measurements. Total soluble solids (TSSs) were determined with a digital refractometer (PR-100, Atago, Tokyo, Japan) and expressed as a percentage. For titratable acidity (TA), 5 mL of juice was titrated with 0.1 N NaOH to a pH of 8.2, using a potentiometer (Hi99301, Hanna Instruments, Woonsocket, RI, USA). The acidity was expressed as a percentage of malic acid (meq: 0.067), calculated by the formula described in AOAC official method 942.15 [61]. The TSSs/TA ratio was calculated by the relation between TSSs and TA.

### 4.3. Analysis of Bioactive Compounds

These analyses were carried out after storage at 30 and 40 days, plus the marketing period, for both maturity stages. Ten representative fruits were crushed by replication in liquid nitrogen and stored in Falcon tubes at −80 °C for further analysis.

#### 4.3.1. Total Anthocyanin Concentration (TAC)

The concentration of anthocyanins was determined by the differential pH method according to Lee et al. [62]. Five g of sample was weighed and extracted in 10 mL of acidified methanol with 1% HCl. The mixture was stirred for 60 s at 5 °C and centrifuged at 5.886× *g* for 10 min at 4 °C. The supernatant was filtered, and the extraction process was repeated with an additional 10 mL of acidified methanol, obtaining a total of 15 mL of extract. One hundred μL were taken from each sample, divided into two aliquots that were added in triplicate in 96-well microplates, each diluted in 200 μL of buffer. Absorbance was measured at 510 and 700 nm in a spectrophotometer (ASYS, UVM340, Biochrom, Cambridge, UK). TAC was expressed in mg of cyanidin-3-O-glucoside equivalents (C3G) 100 g^−1^ FW, calculated using the following formula:

TAC = ((A × MW × DF × 1000))/ε

where A is the difference in absorbance between pH 1 and pH 4.5; MW is the molecular weight of cyanidine 3-O-glucoside; DF is the dilution factor; and ε is the molar coefficient of extinction (20,900 L mol^−1^ cm^−1^).

#### 4.3.2. Total Polyphenol Concentration (TPC)

For phenolic concentration, 5 g of sample was weighed and extracted in 10 mL of 80% methanol, stirring for 5 min. The mixture was stored at 4 °C in darkness for 24 h and then centrifuged at 5.886× *g* for 10 min at 4 °C. The supernatant was filtered, and the precipitate was reextracted with an additional 10 mL of methanol (80%), obtaining 15 mL of extract. The total concentration of phenols was determined by the method of Singleton and Rossi [63], adding 200 μL of the extract to 800 μL of distilled water in an amber Eppendorf tube. Subsequently, in a 96-well microplate, 25 μL of the sample, 50 μL of the Folin–Ciocalteau reagent (10%), and 150 μL of a sodium carbonate solution (770 mM) were mixed. The plate was incubated in the dark at room temperature for 60 min, and the absorbance was measured at 765 nm (ASYS, UVM340, Biochrom, Cambridge, UK). The results were expressed as mg gallic acid equivalents (GAE) 100 g^−1^ FW by a calibration curve.

#### 4.3.3. Antioxidant Capacity by FRAP Method

Antioxidant activity by ferric reducing power (FRAP) was evaluated according to Benzie et al. [64]. A solution of 40 mM HCl, 300 mM acetate buffer, 20 mM ferric chloride, and 10 mM TPTZ (2,4,6-tri(2-pyridyl)-s-triazine) was prepared. FRAP reagent was prepared by mixing 20 mL of acetate buffer, 2 mL of ferric chloride, and 2 mL of TPTZ and allowed to stand at room temperature for 30 min. In an amber tube, 200 μL of extract was mixed with 800 μL of distilled water; then, in a 96-well microplate, 20 μL of the extract and 180 μL of FRAP reagent were added. The absorbance was measured at 593 nm (ASYS, UVM340, Biochrom, Cambridge, UK) after stabilization. Results were expressed as mg Trolox equivalents (TEs) 100 g^−1^ FW, using a Trolox calibration curve.

#### 4.3.4. Antioxidant Capacity by DPPH Method

Antioxidant activity by DPPH was measured following the methods of Blois [65] and Brand-Williams et al. [66]. A DPPH solution was prepared by dissolving 78.86 mg of the reagent in a 500 mL flask with methanol, protected from light. Fifty µL of the sample was mixed with 200 µL of the DPPH solution in a 96-well microplate. The plate was incubated at room temperature for 2 h, and the absorbance was measured at 517 nm in a spectrophotometer (ASYS, UVM340, Biochrom, Cambridge, UK). Results were expressed as mg Trolox equivalents (TEs) 100 g^−1^ FW of cherries, calculated from a Trolox calibration curve.

### 4.4. Statistical Analysis

A completely randomized design with a 2 × 5 factorial structure was used, where the first factor corresponded to the maturity stage (2 levels) and the second factor considered was gas concentrations (5 levels). Due to the destructive methods used for the evaluations, 30 + 4 and 40 + 4 days were analyzed independently. The data expressed as percentages (0–100%) were treated using an angular transformation to stabilize and approximate the variance to a normal distribution. Statistical analyses were performed on the transformed data, and the results were shown in original forms. For each of the response variables, the data were analyzed using mixed linear models (MLMs), after verifying compliance with the assumptions of variance homogeneity and error normality. In cases where significant effects were detected, either by interaction or by individual action of the factors, Fisher’s multiple comparison test (MCT) was applied, with a significance level of 5% (α = 0.05). All statistical analyses were performed using InfoStat software (version 2008) and the R programming language (version 2.7.0).

## 5. Conclusions

Storing Regina cherries under controlled atmosphere (CA) conditions with 10–15% CO_2_ and 10% O_2_ at 0 °C for 30–40 days, followed by 2 days at 5 °C and 2 days at 10 °C, has proven to be highly effective in preserving postharvest quality. These treatments significantly reduced internal browning, decay incidence, and visual quality loss while maintaining lightness, acidity levels, soluble solids, anthocyanin and polyphenol concentrations, and antioxidant capacity. The use of moderate to high CO_2_ combined with moderate O_2_ levels offers a realistic and effective alternative for extending the shelf life of Regina sweet cherry, avoiding the risk of extreme atmospheres. Additionally, harvesting the fruit at the Light Mahogany maturity stage provided further advantages, as it preserved quality during storage and commercialization, enhancing consumer acceptance. These results suggest that designing specific storage protocols for the Regina cv., considering different maturity stages and gas atmospheres, is key to extending its commercial shelf life and the overall quality for distant markets, consolidating its potential competitiveness for the fruit export industry.

## Figures and Tables

**Table 1:** Pedicel dehydration and decay of Regina sweet cherries after 30 and 40 days of storage at 0 °C, followed by 2 days at 5 °C and 2 days at 10 °C (30 + 4 d and 40 + 4 d), analyzed according to independent factors.

Factors	30 + 4 d (%)		40 + 4 d (%)	
	W/PD (%) ^3^	Decay (%)	W/PD (%)	Decay (%)
Maturity stage ^1^	n/s	n/s	n/s	n/s
LM	92.53	0.54	89.80	1.50
DM	92.53	0.52	84.73	1.22
Gas atmosphere (CO_2_ + O_2_) ^2^	n/s	*	n/s	*
15 + 10	89.17	0.27 b ^4^	86.67	0.34 b
10 + 10	90.83	0.08 b	87.50	0.70 b
10 + 5	93.33	0.00 b	85.83	0.26 b
5 + 5	93.33	0.86 a	86.67	2.63 a
MA-4 + 16	96.00	1.45 a	89.67	2.88 a

^1^ LM: Light Mahogany, DM: Dark Mahogany; ^2^ CO_2_ (%) + O_2_ (%): 15 + 10, 10 + 10, 10 + 5, 5 + 5, 4 + 16; ^3^ Acceptable pedicel dehydration or no pedicel dehydration; ^4^ Different lowercase letters in the vertical direction indicate statistically significant differences between factor levels independently (maturity stage and storage atmosphere). * Statistically significant differences were observed in at least one of the means of the factor levels, according to Fisher’s multiple comparison test (*p*-value < 0.05); n/s indicates not significant.

**Table 2:** Browning index of Regina sweet cherries at the beginning (initial control) and after 30 and 40 days at 0 °C, followed by 2 days at 5 °C and 2 days at 10 °C (30 + 4 d and 40 + 4 d), analyzed according to independent factors.

Factors	Browning Index		
	Initial	30 + 4 d	40 + 4 d
Maturity stage ^1^	n/s	n/s	*
LM	0.00	20.55	21.60 b
DM	0.00	19.50	26.70 a
Gas atmosphere (CO_2_ + O_2_) ^2^		*	*
15 + 10	na	15.38 b ^3^	19.63 c
10 + 10	na	16.63 b	21.38 c
10 + 5	na	21.75 a	22.38 c
5 + 5	na	24.88 a	26.25 b
MA-4 + 16	na	21.50 a	31.13 a

^1^ LM: Light Mahogany, DM: Dark Mahogany; ^2^ CO_2_ (%) + O_2_ (%): 15 + 10, 10 + 10, 10 + 5, 5 + 5, 4 + 16; ^3^ Different lowercase letters in the vertical direction indicate statistically significant differences between factor levels independently (maturity stage and gas atmosphere). * Statistically significant differences were observed in at least one of the means of the factor levels, according to Fisher’s multiple comparison test (*p*-value < 0.05); n/s indicates not significant; na, not applicable.

**Table 3:** Firmness parameters of Regina sweet cherries at the beginning (initial control) and after 30 and 40 days at 0 °C, followed by 2 days at 5 °C and 2 days at 10 °C (30 + 4 d and 40 + 4 d), analyzed according to independent factors.

Factors	Firmness					
	Initial		30 + 4 d		40 + 4 d	
	gf mm^−1^	°Ush	gf mm^−1^	°Ush	gf mm^−1^	°Ush
Maturity stage ^1^	n/s	n/s	*	*	*	n/s
LM	326.47	79.03	313.43 a ^3^	86.67 a	288.13 a	78.33
DM	305.67	78.15	284.93 b	79.88 b	251.08 b	78.79
Gas atmosphere (CO_2_ + O_2_) ^2^			n/s	*	*	*
15 + 10	na	na	304.19	83.35 a	254.09 b	76.22 b
10 + 10	na	na	295.68	83.33 a	278.53 a	79.73 a
10 + 5	na	na	294.44	84.26 a	271.65 ab	75.43 b
5 + 5	na	na	302.94	84.17 a	260.04 b	80.09 a
MA-4 + 16	na	na	298.65	81.03 b	283.71 a	81.33 a

^1^ LM: Light Mahogany, DM: Dark Mahogany; ^2^ CO_2_ (%) + O_2_ (%): 15 + 10, 10 + 10, 10 + 5, 5 + 5, 4 + 16; ^3^ Different lowercase letters in the vertical direction indicate statistically significant differences between factor levels independently (maturity stage and gas atmosphere). * Statistically significant differences were observed in at least one of the means of the factor levels, according to Fisher’s multiple comparison test (*p*-value < 0.05); n/s indicates not significant; na, not applicable.

**Table 4:** Internal flesh and external skin color parameters of Regina sweet cherries, obtained in the initial evaluation, between maturity stages.

Factor	Internal Flesh Color			External Skin Color		
	L_i_	C_i_	H_i_	L_e_	C_e_	H_e_
Maturity stage ^1^	*	*	*	*	*	*
LM	29.50 a ^2^	44.67 a	34.13 a	32.04 a	31.46 a	16.06 a
DM	25.70 b	39.89 b	27.17 b	26.98 b	16.93 b	14.88 b

^1^ LM: Light Mahogany, DM: Dark Mahogany; ^2^ Different lowercase letters in the vertical direction indicate statistically significant differences in factor maturity stage. * Statistically significant differences were observed, according to Fisher’s multiple comparison test (*p*-value < 0.05).

**Table 5:** Total soluble solids (TSSs), titratable acidity (TA), and TSS/TA ratio of Regina sweet cherries at the beginning of storage between maturity stages.

Factor	TSSs (%)	TA (%)	TSSs/TA
Maturity stage ^1^	*	*	*
LM	19.70 b ^2^	0.82 a	24.15 b
DM	21.33 a	0.67 b	32.15 a

^1^ LM: Light Mahogany, DM: Dark Mahogany; ^2^ Different lowercase letters in the vertical direction indicate statistically significant differences for maturity stage. * Statistically significant differences were observed in at least one of the means of the factor levels, according to Fisher’s multiple comparison test (*p*-value < 0.05).

**Table 6:** Total soluble solids (TSSs), titratable acidity (TA), and TSSs/TA ratio of Regina sweet cherries by treatment, after 30 days at 0 °C + 2 days at 5 °C + 2 days at 10 °C analyzed based on the interaction between factors.

Treatments ^1,2^	TSSs (%)	TA (%)	TSSs/TA
LM-15 + 10	17.03 f ^3^	0.59 a	28.77 e
LM-10 + 10	17.83 e	0.58 a	30.80 d
LM-10 + 5	18.73 c	0.50 c	37.60 b
LM-5 + 5	18.03 d	0.49 c	36.87 b
LM-MA 4 + 16	16.33 g	0.55 b	29.77 de
DM ^2^-15 + 10	19.73 a	0.58 a	34.30 c
DM-10 + 10	20.03 a	0.56 b	35.60 b
DM-10 + 5	19.07 b	0.53 b	36.37 b
DM-5 + 5	19.97 a	0.57 a	35.07 c
DM-MA 4 + 16	20.07 a	0.43 d	46.73 a
Significance	*	*	*

^1^ LM: Light Mahogany, DM: Dark Mahogany; ^2^ CO_2_ (%) + O_2_ (%): 15 + 10, 10 + 10, 10 + 5, 5 + 5, 4 + 16; ^3^ Different lowercase letters in the vertical direction indicate statistically significant differences between treatments. * Statistically significant differences were observed in at least one of the means of the factor levels, according to Fisher’s multiple comparison test (*p*-value < 0.05).

**Table 7:** Total soluble solids (TSSs), titratable acidity (TA), and TSSs/TA ratio of Regina sweet cherries by treatment, after 40 days at 0 °C + 2 days at 5 °C + 2 days at 10 °C analyzed according to independent factors.

Factors	TSSs (%)	TA (%)	TSSs/TA
Maturity stage ^1^	n/s	n/s	*
LM ^1^	17.29	0.47	37.00 b
DM	17.84	0.44	40.91 a
Gas atmosphere (CO_2_ + O_2_) ^2^	*	*	*
15 + 10	18.82 a ^3^	0.44 a	43.17 a
10 + 10	17.53 a	0.49 a	35.80 c
10 + 5	16.08 b	0.48 a	33.53 c
5 + 5	17.9 a	0.46 a	38.80 b
MA-4 + 16	17.45 a	0.40 b	43.47 a

^1^ LM: Light Mahogany, DM: Dark Mahogany; ^2^ CO_2_ (%) + O_2_ (%): 15 + 10, 10 + 10, 10 + 5, 5 + 5, 4 + 16; ^3^ Different lowercase letters in the vertical direction indicate statistically significant differences between factor levels independently (maturity stage and gas atmosphere). * Statistically significant differences were observed in at least one of the means of the factor levels, according to Fisher’s multiple comparison test (*p*-value < 0.05); n/s indicates not significant.

**Table 8:** Treatments based on maturity stage and gas atmosphere composition.

Treatments	Maturity Stages	CO_2_ (%)	O_2_ (%)
LM-15 + 10	Light Mahogany ^3^	15	10
LM-10 + 10	Light Mahogany	10	10
LM-10 + 5	Light Mahogany	10	5
LM-5 + 5	Light Mahogany	5	5
LM-MA ^1^ 4 + 16 (C) ^2^	Light Mahogany	3 to 4	16 to 17
DM-15 + 10	Dark Mahogany ^4^	15	10
DM-10 + 10	Dark Mahogany	10	10
DM-10 + 5	Dark Mahogany	10	5
DM-5 + 5	Dark Mahogany	5	5
DM-MA 4 + 16 (C)	Dark Mahogany	4	16

^1^ Modified atmosphere; ^2^ Control; ^3^ LM; ^4^ DM.

## Data Availability

The original contributions presented in this study are included in the article. Further inquiries can be directed to the corresponding author.
